# Theory in Practice instead of Theory versus Practice – Curricular design for Task-based Learning within a competency oriented Curriculum

**DOI:** 10.3205/zma000946

**Published:** 2015-02-11

**Authors:** Thomas Rotthoff, Matthias Schneider, Stefanie Ritz-Timme, Joachim Windolf

**Affiliations:** 1 Heinrich-Heine-University Düsseldorf, Medical Faculty, Deanary of study, Düsseldorf, Germany; 2 University Hospital Düsseldorf, Department for Endocrinology and Diabetes, Düsseldorf, Germany; 3 Heinrich-Heine-University Düsseldorf, Deanary of Medical Faculty, Düsseldorf, Germany; 4 University Hospital Düsseldorf, Policlinic for Rheumatology, Düsseldorf, Germany; 5 University Hospital Düsseldorf, Institute for Forensic Medicine, Düsseldorf, Germany; 6 University Hospital Düsseldorf, Department for Trauma- and Handsurgery Düsseldorf, Germany

**Keywords:** Task based learning, learning transfer, knowledge practice, assessment tool, workplace based assessment, undergraduate medical education

## Abstract

**Objective: **Already during their studies, medical students should intensively train their clinical thinking and practice skills, enhancing their clinical expertise in theoretical and practical terms.

**Methods: **Based on the findings of educational research, a new curriculum for clinical training was developed at Duesseldorf University, focussing on workplace-based teaching, learning and assessment.

**Results: **For students in their 3^rd^, 4^th^ and 5^th^ year of study, our curriculum is based on learning with patient complaint items in regard to multidisciplinary areas of outpatient and inpatient care. For this educational format, 123 complaint items were defined and their compatibility with diseases from various disciplines was tested. Based on the complaint of a specific case, students locate the underlying disease pattern, the differential diagnostic and therapeutical procedures and thereby deepen the required knowledge in the basic subjects. Study books have been created by the clinical departments to support this process. Learning is integrated in competence-oriented and workplace-based learning and assessment, offering a close-knit contact between students and doctors.

**Conclusion: **The concept allows the integration of theory into practice and the integration of knowledge from the basic, clinical-theoretical and clinical subjects into clinical thinking and action.

## Zusammenfassung

**Zielsetzung: **Medizinstudierende sollen bereits während ihres Studiums ärztliches Denken und Handeln intensiv trainieren und ihre klinische Expertise in theoretischer und praktischer Hinsicht entwickeln.

**Methodik: **Ausgehend von den Erkenntnissen der Lehr- und Lernforschung wurde ein Curriculum für die klinisch-praktische Ausbildung im Modellstudiengang Düsseldorf entwickelt, welches auf das arbeitsplatzbezogene Lehren, Lernen und Prüfen fokussiert.

**Ergebnisse: **Das Curriculum basiert für Studierende im 3, 4 und 5. Studienjahr wesentlich auf dem Lernen an Behandlungsanlässen von Patienten in multidisziplinären Bereichen der ambulanten und stationären Versorgung. Für dieses Lehrformat wurden 123 Behandlungsanlässe definiert und deren Verknüpfbarkeit mit Krankheitsbildern aus den verschiedenen Fachdisziplinen geprüft. Ausgehend vom Behandlungsanlass eines konkreten Falles, erarbeiten sich die Studierenden das zugrundeliegende Krankheitsbild sowie das differentialdiagnostische und therapeutische Vorgehen und vertiefen dabei das notwendige Wissen in den Grundlagenfächern. Zur Lernunterstützung wurden Studienbücher von den Kliniken erstellt. Das Lernen ist eingebunden in kompetenzorientiertes und arbeitsplatzbezogenes Lernen und Prüfen mit einer intensiven Kontaktzeit zwischen Studierenden und Ärzten.

**Schlussfolgerung: **Das Konzept ermöglicht die Integration von Theorie in die Praxis sowie die Integration von Wissen aus den Grundlagen-, klinisch-theoretischen und klinischen Fächern in das ärztliche Denken und Handeln.

## Introduction

### What is the importance of practice in the study of medicine?

Over the recent years, the often complained lack of practical experience and the „overload“ of theory in studies has led to a greater emphasis on practical content in the curricula of medical studies in Germany. Critics of this development warn against letting the theoretical content become a dangerous lightweight in medical training. The critique on intensifying practical units is probably due to the inconsistent definition of the word „practice“. Concerning the study of medicine, „practice“ is often reduced to learning or to the application of either practical, manual or communication skills. However, in the German language, the term „practice“ also includes the notion of „exercise“ and „experience“ [1], which is why in the medical profession, „practice“ in general means to pursue medical practice in outpatient or inpatient daily work. According to this understanding, „practice“ involves - apart from skills such as medical history taking and physical examination - primarily diagnostic and therapeutic thinking and action for a specific patient case. For this, profound knowledge and scientifically trained thinking is an essential precondition. The cause of these diverse perceptions of the term „practice“ within the study of medicine and the medical profession is also due to the fact that with increasing clinical experience, theory knowledge is often only implicitly integrated into the workflow of a medical workday. Only when issues grow more complex and go beyond the daily routine, requiring a deeper discussion, theory knowledge is explicitly perceived or used [2]. For more complex issues, the linking of knowledge in the basic subjects with clinical knowledge can particularly contribute to a more precise diagnosis [3]. This integration of knowledge into daily medical practice is not yet available for medical students because they are lacking clinical knowledge and its application - in brief: the clinical expertise [4]. Among others, clinical expertise arises from elaborating patient cases and comparing them with previous ones [2]. During their studies, students normally have few opportunities to develop their clinical expertise by means of a reflected and guided discussion on specific patient cases. Thus, they initially access the cases with their knowledge of the theoretical (basic) subjects [4]. Therefore, a practical medical education requires the support of students in developing their expertise by integrating their knowledge from the basic, clinical-theoretical and clinical subjects into the clinical thinking and acting in specific patient cases. How can this training be successfully implemented into medical studies? Is it sufficient to more often delegate students into hospitals or doctors‘ offices, also on behalf of the increasing shift of medical care into the outpatient sector, as proposed for example by the National Association of Panel Physicians in Germany [http://www.kbv.de/html/4473_333.php [cited 2013 Nov 28]. The German Medical Licensure Act (ÄAppO) [http://www.gesetze-im-internet.de/_appro_2002/BJNR240500002.htm cited 2013 Nov 28] denominates various formats of practical training: a four-months clerkship (Famulatur), same as five block courses of 1-6 weeks duration for differential diagnosis and therapy of the most common diseases under clinical conditions of internal and outpatient daily practice. An additional training format are 476 hours of bedside teaching, of which 50% must be performed in the ration of one teacher to maximum three students and the other 50% as a presentation of a patient with one teacher for maximum six students. As for the clerkship - the German word „Famulatur“ is derived from Latin „famulus = learner“ - the ÄAppO does not specify any educational format or content and thus gives plenty of rope for individual learning. The own experience and the perception of present students doubtlessly substantiate a considerable increase in learning efficiency during a Famulatur. However, the success of studying is also determined by factors that require structuring of learning. This is not automatically given in a clerkship. These factors particularly include student-centered learning, feedback, stimulation of complex thinking processes, learning support, a high frequency of assessment same as empathy and appreciation [5], [6]. Although self-directed learning is regarded as an axiomatic goal in adult education, there are also concerns about whether self-directed learning is always expedient. Could a guided and self-directed learning possibly be even more efficient [7]? This is relevant in so far as self-directed learning is established during the process of academic training [8]. The Duesseldorf Curriculum designed a concept for clinical practice training during the academic years 3, 4 and 5, thus offering students the opportunity to develop and enhance clinical expertise already during their medical studies.

## Methods/Development of the project

Theory and practice are integrated by including findings from educational research. The concept design incorporates factors for academic success, same as other findings from educational research.

The following points have been implemented since the winter term 2013/14:

- Case-based learning on the patient, starting from a complaint or consultation issue in multidisciplinary areas of outpatient and inpatient care [9], [10], [11]
- Supply of material for learning support (so-called Study Books) [12]
- Regular and structured feedback [5]
- Competency-oriented [13], [14] and workplace-based assessment [15]
- Fostering the formation of identity from student to graduate [16]
- Promotion of the student‘s own learning technique and learning progress towards a more profound learning [17]
- Increase of contact between students and physicians in terms of a role model [18]

For case-based learning, 123 complaint items were defined. Initially, the Working Committee of the Curriculum Reform created a synopsis out of three catalogs: the task list of the University of Dundee (UK) [11], the German Catalog of Learning Objectives (GK2-2009) and the Dutch Blue Print 2009 [19].

Ab initio, this development involved student representatives as members of the Committee. Directors and lecturers of 44 clinics and institutes of Duesseldorf‘s University Hospital participated in analyzing and revising the allocation of substantial diseases / themes to the Duesseldorf List of Complaints. This list was subsequently passed by the Faculty Council. On one hand, the List of Complaints had to be extensive in order to enable a link to all the diseases that are relevant for medical studies in various disciplines. On the other hand, the list had to be limited in order to provide practical use in the clinical practice training. The handling of the complaints is largely independent of the health care environment. For example, both „abdominal pain“ and „shortness of breath“ are part of outpatient primary care, same as of any other level of health care. The underlying diseases or disorders of these complaints, however, may vary within their health care environment, implicating different diagnostic and therapeutic approaches. Reasons for consultation that occur predominantly in outpatient and primary care, such as „advisory service for healthy individuals and for parents of healthy children“ or „early detection / screening“ or „vaccination“, were also taken into account. The Duesseldorf List of Complaints and Reasons for Consultation was broadly adopted into the current draft of the National Competency-Based Learning Catalog for Medicine (NKLM) (see Table 1 (Tab. 1)).

### Curricular Implementation

In each semester of their 3^rd^ year, the students go through a four-week practice block and a four-week study block. In their 4^th^ and 5^th^ year, they enroll in two of each blocks per semester. Within the practice blocks, the focus lies on learning in actual patient cases. The study blocks complement the case-based learning by providing systematic knowledge in seminars and lectures. The last week of each study block is free of courses, giving the students room for profound individual studies. This week is concluded by an interdisciplinary examination. 

**Figure 1:**
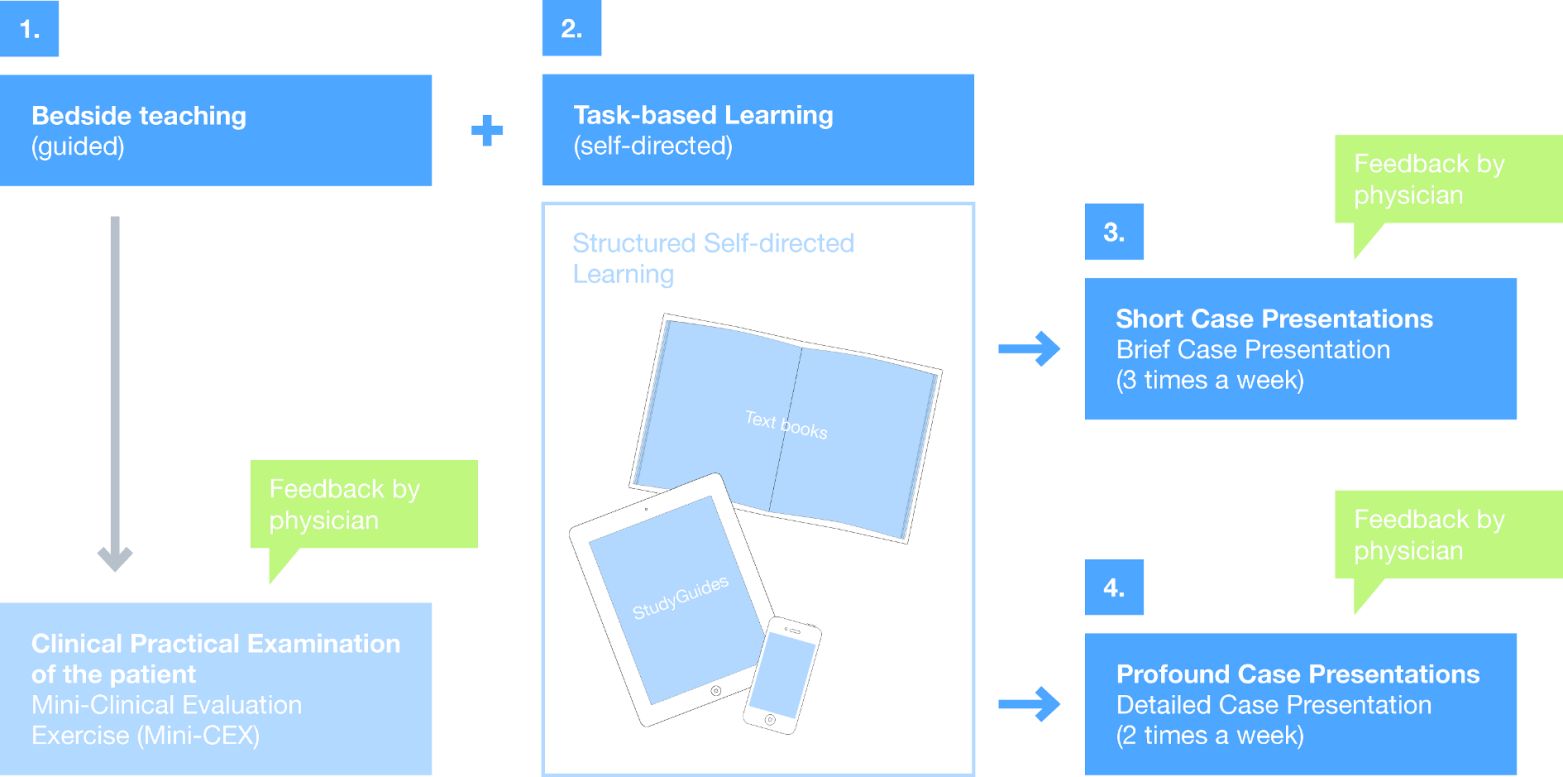
Formats of Learning and Assessment within practice blocks (a week´s example)

Within the ten four-week practice blocks, the students complete the internships that are required by the ÄAppO. The structured clinical practice training takes place here and the focus lies on the integration of theory and practice by means of case-based learning (see Figure 1 (Fig. 1)). In addition to bedside-teaching lead by a doctor or health care professional, students achieve relevant learning objectives and depict diseases on specific patients. Commencing with the particular patient‘s complaint or reason for consultation, the students then exemplarily elaborate the differential diagnostic and therapeutic procedures. This educational format, rooted in the so-called „Task-based Learning“, supports the acquisition of clinical knowledge and funds the student‘s self-responsibility [11].

Students are requested to elaborate at least 80% of the 123 complaints and reasons for consultation during the overall 40 weeks of practice blocks. Basically it is even allowed to repeatedly elaborate the same complaint item, as a complaint can be based on various diseases from different disciplines. Moreover, a complaint can vary in every individual patient‘s case, depending on the interaction with further comorbidities.

When students encounter a patient with one of the complaints or reasons for consultation mentioned in the Duesseldorf List (e.g. memory impairment, difficulty in breathing, incidental finding during diagnostic process, etc.), they are able to elaborate the underlying disease and to establish the differential diagnostic and therapeutic procedures by regarding the complaint item and relating it to the particular case. In doing this, the students can expand the required knowledge in the basic subjects. In order to support the learning process and in contrast to the clerkships (Famulatur), the practice blocks are structured as follows:

1. **Patient Contact: **For the duration of one week, the students are assigned to their patients with accordant complaints or reasons for consultation by the doctors of the clinical departments and institutes. The students contact „their“ patient, take a history, perform a physical examination and do a research based on the patient‘s file. Subsequently, they elaborate the required knowledge by means of textbooks or internet portals and with the support of study guides (see below). In the following week of the practice block, the students revolve to another clinical department or institute.
2. **Study Guides: **Study Guides were developed by Duesseldorf‘s clinical departments and institutes in order to support learning. The Guides contain chapters with a selection of complaint items or other reasons for consultation relevant to each specific discipline. With the complaint „shortness of breath“, for example, the students can use a web-based learning platform to find the Study Guides created by those clinical departments and institutes that have selected this complaint item as a course content and have created a corresponding chapter. These disciplines explain the significance of the complaint „shortness of breath“ from their subject-specific point of view. In this context, all Study Guides adhere to a conceptual framework: a) learning objectives form the subject‘s point of view, b) diseases matching the complaints, c) special features in clinical management d) literature recommendations for further reading, e) questions for reflection. It is especially the informations on the special features in clinical management and the questions for reflection that exceed the contents of textbooks.
3. **Case Documentation and Case Presentation**: Using predetermined questions (see Table 2 (Tab. 2)), students document every case (3-4 cases per week) and present it to a doctor within the clinical workplace. The brief case presentation is assessed by the instructor by means of a checklist, taking into consideration the presentation of the case, the critical analysis of findings and diagnostic procedures, the elaboration of pros and cons of the applied therapy as well as suggestions for improvements in patient management. The students receive an individual feedback on this.
4. **Case presentation in the tutorial:** In addition, tutorials with a maximum of 15 students take place twice a week, requesting its attendants to present their cases in detail and furthermore discuss and reflect these cases with their fellow students. The members of a tutorial group go through various clinical rotations on a weekly basis, but however remain within their group for at least one semester. This concept was chosen for group dynamic reasons in order to enable the group‘s continuous development process within a familiar constellation. A grouping with students of the same clinical rotation would imply a weekly change of group members in the tutorial. In consultation with student representatives, the consistency of the group was classified as a major target. Over the course of ten practice blocks, the students will work out a total of 160 case studies, regarding the complaint items from the perspective of various disciplines. The presenting students of the group take the „expert role“, i.e. they respond to specific inquiries and moderate the discussion, which at the same time requires and stimulates a profound preparation and analysis of the subject matter. Each tutorial is supported by a medical tutor who should preferably accompany the group over the entire four-week period of a practice block. The case presentations are considered as assessments and are rated by the medical tutors in regard of the structure and content of the presentation, facilitation of the group discussion and the reflexions made on the individual learning progress and patient management.
5. **Clinical practical examinations (Mini-Clinical Evaluation Exercise, Mini-CEX): **At least once per practice block, each student has to pass a clinical-practical examination that is predominantly performed on the patient. The students are given notice of the potential exam issues at the beginning of a practice block‘s week. The contents of the examination are usually procured during bedside teaching by the responsible clinical department or institute. The needs and expectation for the Mini-CEX and the rating criteria are transparent and available for the students. A part of the examinations can be practiced in advance in the skills lab. About 80 Mini-CEX that have been developed by the clinical departments and institutes are currently available. The range of topics includes various tests on medical skills such as physical examination techniques, change of postoperative bandaging, Prick testing, bedside tests on blood transfusions or communication skills in dealing with suicidality in a patient conversation. The majority of the examinations take place at the bedside. Some manual skills, such as „implementation of a knee aspiration“ (Orthopedics) are performed on a model, whereas more complex communicative situations such as the „prevention of post-traumatic stress disorder“ (Psychosomatic Medicine and Psychotherapy) are performed with simulated patients. The time frame of a Mini-CEX should average about 20-30 minutes, including an oral feedback.

**Figure 2:**
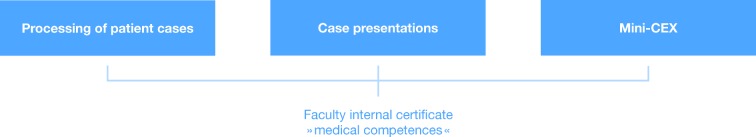
Examination framework within practice blocks

All of the above listed assessments of point 3) to 5) have to be successfully passed, they sum up to the faculty internal transcript „Medical Expertise“ (see Figure 2 (Fig. 2)). The student collects them in a portfolio until the point of the Second State Examination (M2). 

## Discussion

The integration of theory into practice, as implemented by the Duesseldorf Curriculum, enables the student to develop a clinical expertise and further physical competencies already during their medical studies. In addition to the acquisition of knowledge, there are diagnostic and therapeutic decision making processes, communication skills, presentation and moderation skills that aim to be trained, same as there are social and ethical issues that should be discussed and deliberate thinking that should be encouraged. A critical regard on the professional medical practice in specific patient cases and the consideration of evidence-based medicine should nurture the students‘ scientific thinking. Our initial experience with the implementation of this concept shows that the present socialization of the students seems to be predominantly characterized by the dualistic terms „true or false“. Dealing with one‘s own nescience, but also with the white spots and uncertainties of modern medicine, is yet unfamiliar to the students, leading to confusion and, for some students, even to a hostile attitude towards the new concept. Similar attitudes can also be observed in some medical tutors, whose teaching now no longer derives from their expert position.

Furthermore, abandoning a traditional subject sequence in combination with interdisciplinary thinking and studying leads to confusion and hostile reactions in some students. For some clinical directors, there is still a major concern about the lack of proper visibility for their discipline in an integrated curriculum. However, it is already apparent that within the practice blocks, students do indeed perceive and evaluate a clinical department‘s or institute‘s good teaching. Clinical departments and institutes that offer a greater involvement of students into clinical practice, providing a valuable clinical practical education, already register an increasing number of applicants for doctoral research studies, clerkships and medical assistants. The new curriculum concept requires a continuous development of the teaching and learning methods within the faculties, aiming at a constructively criticizing and more dynamic interaction between lecturers and students, just as it is presented in feedback encounters. In our view, these changes are the greatest challenge for the future performance and for the further successful implementation of the concept. For the faculties, this requires intensive communication within and between the involved pressure groups. In addition to the numerous past and future lecturer trainings for the above mentioned teaching and examination formats, specific on-site coaching in clinical departments and institutes are provided by Masters of Medical Education (MME). Tutorial videos for the various teaching and examination formats are already available and are constantly expanded in collaboration with student representatives.

The intense contact with patients, the self-contained case studies and the case discussions are apt to enhance the learner‘s development from a student towards a medical doctor. The close interaction with medical colleagues in the clinic‘s work environment also enables the students a significant peer on role models [18]. Students report on decreasing restraints and fears in contact with patients. 

The presented structure of studying in a clinical environment with complex requests for various medical skills requires a scientific evaluation with quantitative methods, e.g. for a longitudinal analysis of the latest examination results, same as qualitative evaluation methods like focus groups and interviews, etc. for analyzing the learning behavior during task-based learning. The curriculum requires the provision of resources. With an intake of 12 students into a clinical department or institute, 36 hours of bedside teaching and an additional 4 hours of tutorials must be performed per week, not taking into consideration the additional hours for seminars and lectures for the study blocks. With this investment, an academic medical education allows the integration of theory into practice, same as the integration of knowledge from the basic, clinical-theoretic and clinical subjects into medical thinking and action.

## Acknowledgement

We thank all members of the team for curriculum development: Ellen Bauchrowitz; Dr. Hans-Martin Bosse MME; Eva Bramann, Aurèle Comparot; Prof. Dr. Walter Däubener; Prof. Dr. Ulrich Decking (stellv. Studiendekan); Babette Dufrenne, M.A; Dr. Urte Fahron; Dr. Lars Galonska; PD Dr. Matthias Hofer, MME; Prof. Dr. Alfons Hugger, MME (stellv. Studiendekan Zahnmedizin), Pascal Kalbhen; Dr. André Karger; Julia Karthein; Dr. Alexandra Kravchenko, Prof. Dr. Klaus-Dietrich Kröncke, MME; Malte Kohns; Christian Michalek, M.A; Dr. Anja Nilges, Univ.-Prof. Dr. Harald Rieder; Caroline Rump, Dr. Anja Vervoorts; Dr. Simone Weyers, MME.

We also thank Doris Nord for proofreading the English version of the manuscript and Benjamin Brinkmann for processing the figures.

## Competing interests

The authors declare that they have no competing interests.

## Figures and Tables

**Table 1:**
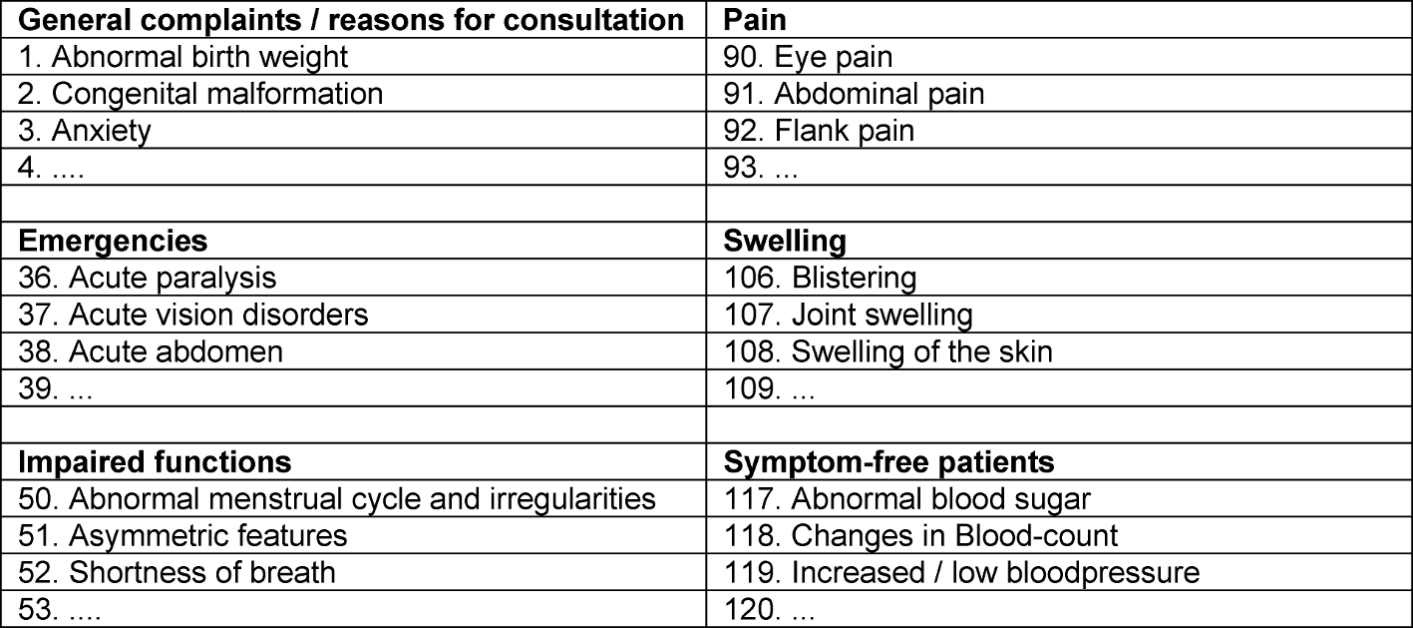
Reasons for Consultation

**Table 2:**
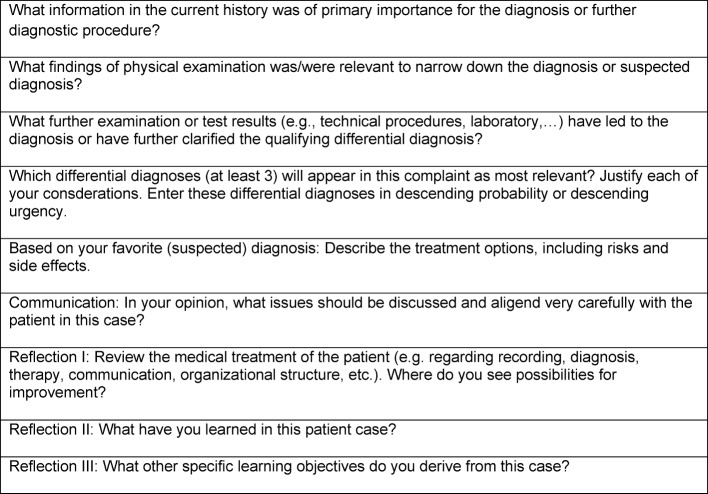
Tasks for documentation of patient case
